# Spotlight on pomalidomide: could less be more?

**DOI:** 10.1038/leu.2017.156

**Published:** 2017-06-16

**Authors:** T Zander, S Aebi, T Pabst, C Renner, C Driessen

**Affiliations:** 1 Department of Medical Oncology, Luzerner Kantonsspital, Lucerne, Switzerland; 2 Department of Medical Oncology, University Hospital Berne, Berne, Switzerland; 3 Onkozentrum Hirslanden, Zurich, Switzerland; 4 Department of Oncology and Hematology, Kantonsspital St. Gallen, St. Gallen, Switzerland

Multiple myeloma (MM) is among the most frequent hematologic malignancies. Despite recent treatment advances, MM remains an incurable disease in the vast majority of cases. The course of the disease is characterized by multiple relapses.

Pomalidomide is a third-generation, oral immunomodulatory drug with activity in patients with relapsed and refractory MM. The pivotal MM-003 trial (pomalidomide 4 mg per day d1−21 q28 + low dose dexamethasone (LoDEX) vs high dose dexamethasone) showed improvement in median progression-free survival (PFS) from 1.9 months with LoDEX to 4.0 months with pomalidomide/LoDEX.^1^ A preplanned interim analysis of overall survival (OS) showed superiority of pomalidomide. Toxicity of pomalidomide in the MM-003 trial, however, was considerable, with 60% of patients experiencing drug-related G3/4 toxicity. Neutropenia (48 vs 16%) and pneumonia (13 vs 8%) were significantly more common in the pomalidomide arm. This resulted in frequent dose interruptions (67%) and dose reductions (27%). This suggests that for the majority of patients the 4 mg daily dosing schedule (4 mg daily on 21 of 28 days) is too toxic, and that strategies to deliver reduced dosing of pomalidomide are of high practical relevance.

The drug costs of pomalidomide are high even for healthcare systems in developed countries (Switzerland: 12.400 CHF per 4 week cycle; US: US$ 13.700 per 4 week cycle). Interestingly, the manufacturer chose a pricing model that is independent from the capsule strength (costs for one capsule 1 mg=2 mg=3 mg=4 mg). In patients requiring dose reductions due to hematologic toxicity, daily dosing of reduced strength pomalidomide (for example, 2 mg daily) is approved and suggested by the manufacturer. This delivers 50% less pomalidomide to the patient, albeit at 100% of the price of full dosing. Given this discrepancy, one may think about alternative strategies, such as delivering the 4 mg capsule strength on an alternate day schedule, which would save 50% of the drug costs compared to daily 2 mg. Obviously, this requires that pomalidomide has adequate pharmacokinetic properties. The evidence supporting the established vs alternative dosing schedules of pomalidomide is, therefore, worth reconsidering.

## Alternative dosing schedules

Due to its pharmacological characteristics, pomalidomide is well suited for alternate day dosing; in contrast to other immunomodulatory drugs (IMiDs), pomalidomide has the longest half-life (t_½_) of all IMiDs with a mean of 7.5 h (t_½_ lenalidomide: 3 h) in patients with MM.^2^

**Figure 1:**
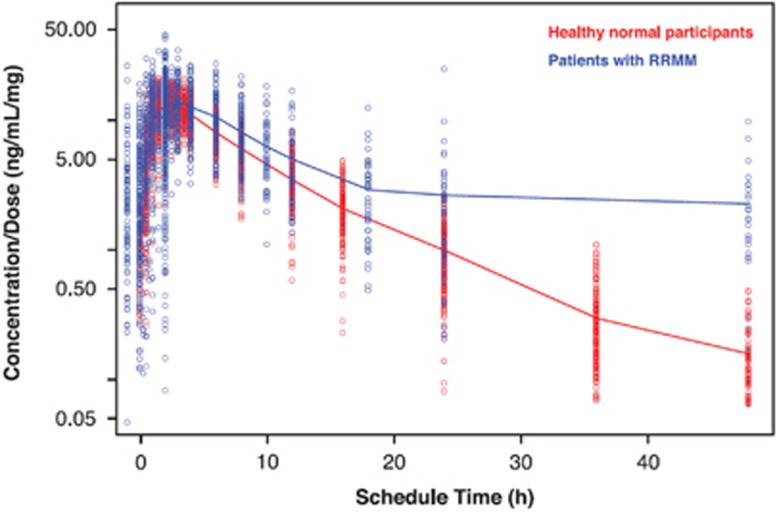
Individual dose-normalized pomalidomide concentration vs time profiles: healthy normal participants vs patients with MM^3^.

In a population pharmacokinetics analysis, Li *et al.*^3^ published a model demonstrating a substantially deeper tissue/organ penetration of pomalidomide in MM patients compared to healthy subjects. The peripheral volume of distribution (V_3_/F) was eightfold higher in patients with active MM (71.5 l) compared with healthy participants (8.5 l). Correspondingly, the largest V_3_/F was found in patients with stage III disease. As a result, the decline of the plasma concentration at the terminal phase was slow (Figure 1). These data make pomalidomide an ideal candidate for alternate day dosing.

The early dose-finding studies with pomalidomide resulted in conflicting data. Schey *et al.*^4^ examined the safety and tolerability of pomalidomide in patients with relapsed refractory MM in the first-in-man phase I clinical trial in 2004. The main objective of the study was to establish the maximum tolerated dose (MTD) of pomalidomide. Seventeen percent (4/24) of the participants developed deep vein thrombosis. The main hematologic dose-limiting toxicity (DLT) was grade 4 neutropenia in patients taking more than 2 mg per day (neutropenia ⩾ grade 3: 100%). MTD in this patient population was therefore defined as 2 mg daily Table 1.

Because of the toxicities observed, the same group undertook a second dose-finding phase 1 study using alternate day administration with the aim to potentially reduce toxicity while maintaining efficacy.^5^ Twenty patients with relapsed myeloma were treated on alternate days. This schedule of pomalidomide was associated with marked reduction of thrombotic episodes (no events observed) and less severe myelosuppression (neutropenia ⩾ grad 3: 45%) while maintaining anti-myeloma activity (>50% partial response or better; 10% complete response). The median OS and PFS were 33 and 10.5 months, respectively. MTD was defined as 5 mg on alternate days.

These trials laid the foundation for subsequent clinical development. Lacy *et al.*^6^ conducted a phase II trial of pomalidomide in combination with dexamethasone in 60 patients (40% with lenalidomide-refractory disease) with less than three prior therapies. Sixty patients received pomalidomide (2 mg daily) with dexamethasone 40 mg weekly. The overall response rate was 63% with 5% of the patients achieving complete response. The most common grade 3 or 4 toxicity was neutropenia in 31% of the patients.

Subsequently, Lacy *et al.*^7^ tested two different schedules in a sequential non-randomized trial (4 mg for a 28/28-day cycle and 2 mg for a 28/28-day cycle) and showed that both dosing levels resulted in similar activity in dual-refractory myeloma patients.^7^ These non-randomized data confirmed the remarkable activity of pomalidomide but, at the same time, suggested that there may be no clinically significant advantage for 4 mg over the 2 mg with daily dosing schedules, while the 2 mg dose appeared to be more tolerable.

The most recent phase 1 study was conducted by Richardson *et al.*^8^ following a standard ‘3+3’ design to determine the MTD of pomalidomide administered on days 1 to 21 daily, on a 28-day cycle in patients with relapsed and refractory MM. No formal DLTs were observed in the first three patients enrolled at the 2 mg dose level. However, one patient discontinued because of thrombocytopenia, and therefore, the investigators agreed to enroll three additional patients in this cohort. Only one of the six patients treated with 2 mg experienced a formal DLT (grade 3 fatigue). The overall response rate seen in this small cohort of only six patients was lower (⩾partial response: 1/6) than in the 4 mg cohort (⩾partial response: 4/14). Obviously, the study was not powered for such a comparison, which is based on very small numbers. The median PFS was 4.6 months for both doses, and the median OS was 18.3 months in the intention to treat population. From these data it was concluded to recommend a dose of 4 mg daily d1−21 q28 for the pivotal phase III trial (MM-003).

**Figure 2:**
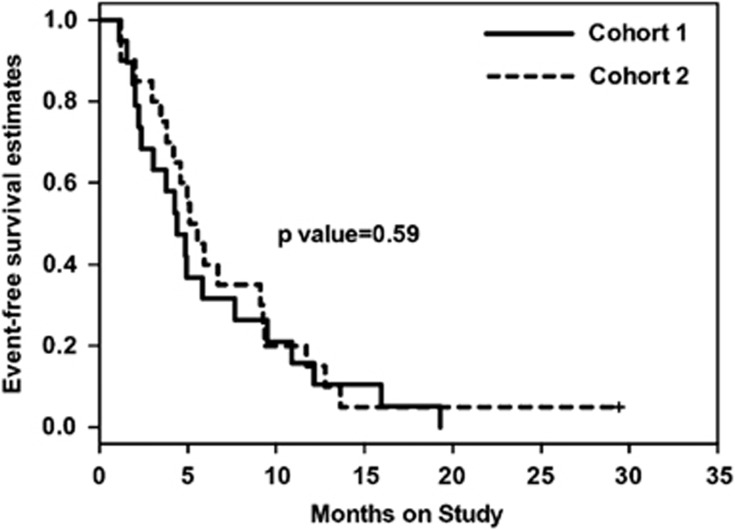
Kaplan−Meier plot comparing event-free survival in the cohort 1 (2 mg 28/28) and 2 (4 mg 21/28).^9^

Very recently, Sehgal *et al.*^9^ revisited the pomalidomide dosing question and evaluated the clinical and pharmacodynamic effects of continuous (cohort 1: 2 mg for 28/28 cycle) or intermittent dosing strategies (cohort 2: 4 mg for 21/28 cycle) of pomalidomide/dexamethasone in patients with lenalidomide-refractory myeloma in a small randomized trial with 39 patients. The cohorts were well balanced. In this study, comparable responses were observed for both dosing schedules with significantly more grade 3/4 side effects in the standard dose cohort (4 mg 21/28). Of note and of critical importance for the treated patients, the event-free survival was not different between both study arms (Figure 2).

Together, these results are consistent with the hypothesis that a lower than the approved standard dosage of pomalidomide (4 mg on 21/28 days) may be sufficient to exploit the anti-myeloma activity of pomalidomide in the clinical setting, while reducing the incidence and severity of side effects.

Looking ahead, the use of pomalidomide for MM, treatment duration and hence pomalidomide-related treatment costs are likely to significantly rise over the next years. The standard pomalidomide schedule currently serves as a backbone in phase III trials for patients with double refractory MM to which novel classes of agents such as monoclonal antibodies are being added in the experimental arm. In particular, the addition of daratumumab might lead to significantly increased treatment durations according to the respective data presented at ASH 2016. Perhaps not surprisingly, this combination with standard dose 4 mg daily pomalidomide caused significant hematological toxicity, with 46% of patients requiring pomalidomide dose reductions.^10^ From this perspective too it appears essential for patients and the healthcare system alike that the pomalidomide backbone therapy is performed with the lowest possible clinical and financial toxicity, while maintaining its clinical efficacy.

Very few trials, sadly, are asking major strategic questions beyond drug approval. One of the big issues today is that the cost of cancer care has increased dramatically, and still few trials are addressing cost effectiveness issues. More bluntly, given that the number of cancer (and myeloma!) patients is predicted to double between 2015 and 2030, and in the light of such significant drug costs, public and academic research likewise must ask whether we can deliver equivalent quality of care and therapeutic efficacy to more patients without a significant increase in financial resources. The best possible use of highly active, but very expensive drugs will be a cornerstone in such a strategy. Pomalidomide might be one good example of how substantial amounts of money may be saved, probably without affecting patient outcome by using a different dose or schedule than in the registration trial. Obviously, the conduct of such dose reduction trials that address the clinical efficacy of more cost-effective treatment schedules are not in the short-term interest of pharmaceutical companies. Such trials are, therefore, only feasible with financial support from academic and public agencies or non-commercial foundations. Indeed, the myeloma research community has a track record in trials that optimized the use of an established drug. For instance, bortezomib is now given subcutaneously^11^ and for elderly patients on a weekly dosing schedule.^12^

We conclude that the optimal dosing regimen of pomalidomide is unclear. The current daily 4 mg treatment standard is based on very little comparative data and raises questions from a toxicity and cost effectiveness perspective, which are highly relevant for our patients and the health care systems worldwide. In our opinion there is a scientific rationale to use pomalidomide on alternate days, especially in patients who need dose reductions or are considered not to tolerate the full registered pomalidomide dose.

In case the data discussed here already prompted clinicians to use an alternate pomalidomide dosing schedule and in the absence of a prospective clinical trial, we propose to collect clinical data and outcome variables of such patients. In addition, the authors would be happy to coordinate such an effort in a framework of an international, prospective cohort study.

## Figures and Tables

**Table 1:** Summary of available clinical efficacy data of pomalidomide in multiple myeloma trials

*Trial*	*Ph*	*N*	*Regimen*	*Dose schedule/MTD*	*ORR %*	*DCR % (⩽SD)*	*Neutropenia*⩾*grade 3*	*PFS; OS (months)*
Schey *et al.*^4^	I	24	Pom	MTD=2 mg per day	54	>90	58% (patients taking > 2mg 100%)	9.7; 22.5
Streetly *et al.*^5^	I	20	Pom ± Dex	MTD=5 mg on alternate days	50	90	45%	10.5; 33
Richardson *et al.*^8^	I	38	Pom ± Dex	MTD=4 mg	21	NA	53% (patients taking > 2mg 60%)	4.6; 18.3
Lacy *et al.*^6^	II	60	Pom/Dex	2 mg (28/28)+DXM	63	88	31%	11.6; 76% at 2 years
								
*Leleu et al.*^13^	II							
Arm A		43	Pom/Dex	4 mg (21/28)+DXM	30	79	65%	NA
Arm B		40	Pom/Dex	4 mg (28/28)+DXM	47	85	58%	NA
								
*Lacy et al.*^7^	II							
Cohort A		35	Pom/Dex	2 mg (28/28)+DXM	49	82	51%	6.5; 78% at 6 months
Cohort B		35	Pom/Dex	4 mg (28/28)+DXM	43	74	66%	3.2; 67% at 6 months
								
*San Miguel MM-003*^1^	III							
Cohort A		302	Pom/Dex	4 mg (21/28)+DXM	21	82	48%	4.0; 12.7
Cohort B		153	Dex	DXM	3	62	16%	1.9; 8.1
								
*Sehgal et al.*^9^	II							
Cohort 1		19	Pom/Dex	2mg (28/28)	21	NA	42%	4.3; 21.7
Cohort 2		20	Pom/Dex	4mg (21/28)	45	NA	45%	5.1; 17.7

Abbreviations: DCR, disease control rate; DXM, dexamethasone; MTD, maximum tolerated dose; ORR, overall response rate; OS, overall survival; PFS, progression-free survival; SD, stable disease.
